# The Feasibility of Metagenomic Next-Generation Sequencing to Identify Pathogens Causing Tuberculous Meningitis in Cerebrospinal Fluid

**DOI:** 10.3389/fmicb.2019.01993

**Published:** 2019-09-03

**Authors:** Shengnan Wang, Yingli Chen, Dongmei Wang, Yongming Wu, Deqiang Zhao, Jianzhao Zhang, Huifang Xie, Yanping Gong, Ruixue Sun, Xifang Nie, Haishan Jiang, Jian Zhang, Wei Li, Guanghui Liu, Xuan Li, Kaibin Huang, Yingwei Huang, Yongjun Li, Hongzhi Guan, Suyue Pan, Yafang Hu

**Affiliations:** ^1^Department of Neurology, Nanfang Hospital, Southern Medical University, Guangzhou, China; ^2^Department of Neurology, Beijing Children’s Hospital, Capital Medical University, Beijing, China; ^3^Department of Neurology, Zhujiang Hospital, Southern Medical University, Guangzhou, China; ^4^Tianjin Medical Laboratory, BGI-Tianjin, BGI-Shenzhen, Tianjin, China; ^5^Department of Infectious Diseases, Nanfang Hospital, Southern Medical University, Guangzhou, China; ^6^BGI Genomics, BGI-Shenzhen, Shenzhen, China; ^7^Department of Neurology, Peking Union Medical College Hospital, Chinese Academy of Medical Sciences & Peking Union Medical College, Beijing, China

**Keywords:** cerebrospinal fluid, *Mycobacterium tuberculosis*, meningitis, metagenomic next-generation sequencing, early diagnosis

## Abstract

**Purpose:**

The application of metagenomic next-generation sequencing (mNGS) in the diagnosis of tuberculous meningitis (TBM) remains poorly characterized. Here, we retrospectively analyzed data from patients with TBM who had taken both mNGS and conventional tests including culture of *Mycobacterium tuberculosis* (MTB), polymerase chain reaction (PCR) and acid-fast bacillus (AFB) stain, and the sensitivity and specificity of these methods were compared.

**Methods:**

We retrospectively recruited TBM patients admitted to the hospital between December 2015 and October 2018. The first collection of cerebrospinal fluid (CSF) samples underwent both mNGS and conventional tests. In addition, patients with bacterial/cryptococcal meningitis or viral meningoencephalitis were mNGS positive controls, and a patient with auto-immune encephalitis was an mNGS negative control.

**Results:**

Twenty three TBM patients were classified as 12 definite and 11 clinical diagnoses, which were based on clinical manifestations, pathogen evidence, CSF parameters, brain imaging, and treatment response. The mNGS method identified sequences of *Mycobacterium tuberculosis complex* (MBTC) from 18 samples (18/23, 78.26%). In patients with definite TBM, the sensitivity of mNGS, AFB, PCR, and culture to detect MTB in the first CSF samples were 66.67, 33.33, 25, and 8.33%, respectively. The specificity of each method was 100%. Among the four negative mNGS cases (4/23, 17.39%), three turned out positive by repeated AFB stain. The agreement of mNGS with the total of conventional methods was 44.44% (8/18). Combination of mNGS and conventional methods increased the detection rate to 95.65%. One patient was diagnosed as complex of TBM and cryptococcal meningitis, in which AFB stain and cryptococcal antigen enzyme immunoassay were positive and the DNA of *Cryptococcus neoformans* was detected by mNGS.

**Conclusion:**

Our study indicates that mNGS is an alternative method to detect the presence of mycobacterial DNA in CSF samples from patients with TBM and deserves to be applied as a front-line CSF test.

## Introduction

Tuberculous meningitis (TBM) is the most devastating manifestation of tuberculosis, with more than half of the patients disabled or died from the disease (Thwaites et al., 2007; Marais et al., 2010). Early diagnosis and prompt treatment have long been recognized as the most important factors to determine the outcome (Thwaites et al., 2013). However, the early diagnosis of TBM remains challenging. The clinical manifestations of TBM are non-specific and may mimic other meningoencephalitis, which can be caused by infectious pathogens including bacteria, virus, fungi, and parasites, leading to difficulty in the determination of the causative organism, especially in the case of sub-acute or chronic infection (Glaser et al., 2006; Solomon et al., 2007; Venkatesan et al., 2013). Early diagnostic identification of precision infectious agents is essential to guide therapy and decrease morbidity and mortality (Studahl et al., 2013). Conventional methods to detect *Mycobacterium (M.) tuberculosis* (MTB) mainly consist of culture and susceptibility testing, microscope cytology, smear microscopy of acid-fast bacilli (AFB), polymerase chain reaction (PCR), real-time PCR to simultaneously detect tuberculosis and rifampicin resistance (Xpert MTB/RIF assay) and immune-assay (Eisenach et al., 1991; Deshpande et al., 2007; Nhu et al., 2014; Brown et al., 2018). Although mycobacterial culture is the gold standard for diagnosis of TBM, low detection rate and the turnaround time of 2–4 weeks limit its use as an early diagnostic selection (Colman et al., 2016). The other microbiological techniques are either time consuming and/or inefficient, resulting in delayed diagnosis and treatment. Therefore, improved techniques capable of detecting infectious pathogens in cerebrospinal fluid (CSF) in a timely manner can help improve the early diagnosis of TBM.

Recently, metagenomic next-generation sequencing (mNGS) has emerged as a sensitive technology capable of detecting pathological organisms from human biopsy samples and body fluid including blood, urine, CSF, and sputum (Wylie et al., 2013; Salzberg et al., 2016; Mai et al., 2017; Parize et al., 2017; Ai et al., 2018; Brown et al., 2018; Guo et al., 2018; Miao et al., 2018). mNGS provides an unbiased assay since it ensures a detailed sequencing of the total DNA or RNA content of the microbiome. Therefore, mNGS has the potential to identify any pathogen in CSF from patients with brain infection to facilitate differential diagnosis (Barzon et al., 2011; Wylie et al., 2013; Chan et al., 2014; Goldberg et al., 2015). Since the first mNGS application in the identification of *Leptospira santarosai* from the CSF in an immunocompromised patient (Wilson et al., 2014), several case reports have successfully utilized mNGS in the diagnosis of viral meningoencephalitis (Perlejewski et al., 2015; Guan et al., 2016), Listeria meningoencephalitis (Yao et al., 2016), and *Vibrio vulnificus* meningoencephalitis (He et al., 2018) from CSF samples. In addition, identification of pathogens by mNGS has been successful in both brain and spinal cord biopsies from 11 patients with suspected brain infection (Salzberg et al., 2016). Recently, a retrospective analysis of CSF mNGS results from 99 pediatric patients with bacterial meningitis indicates 10 different bacteria pathogens identified (Guo et al., 2018). Furthermore, a large-scale prospective study with 511 specimens from infectious patients demonstrates that the sensitivity of mNGS to detect pathogens is higher than cultures and mNGS is less commonly affected by prior antibiotic exposures (Miao et al., 2018). Since mNGS has successfully detected pathogens in the cases of neurobrucellosis (Fan et al., 2018b) and neurocysticercosis (Fan et al., 2018a), it might be used as a front-line or second line diagnostic tool for chronic infectious meningitis especially for undiagnosed cases (Brown et al., 2018). However, mNGS performance with CSF for the detection of MTB still lacks evidence.

Here, we retrospectively reviewed 23 cases of TBM with both mNGS and conventional tests performed in CSF samples, trying to illustrate the feasibility of mNGS in the early diagnosis of TBM and evaluate the sensitivity and specificity among all methods. mNGS identified sequences mapping to DNA of *Mycobacterium tuberculosis complex* (MBTC), including *M. tuberculosis*, *M. africanum*, *M. bovis*, *M. canetti*, and *M. orygis* in 18 cases. mNGS had the highest sensitivity in all methods. Combination of mNGS and AFB stain increased the detection rate. The present study suggests that mNGS could facilitate the identification of MTB in CSF and might be useful as a routine diagnostic procedure for suspected TBM.

## Materials and Methods

### Ethics Statement and Informed Consent

This study was carried out in accordance with the recommendations of guidelines and regulations using human specimen, the institutional review board of Nanfang Hospital, Southern Medical University and Peking Union Medical College Hospital in China, with written informed consent from all patients or their legal representatives. All subjects gave written informed consent in accordance with the Declaration of Helsinki. The retrospective-review protocol was approved by the institutional review board of Nanfang Hospital, Southern Medical University and Peking Union Medical College Hospital in China.

### Participants

All patients were admitted to the Department of Neurology, Nanfang Hospital or Peking Union Medical College Hospital between December 2015 and October 2018. Twenty-three TBM patients with a definite or clinical diagnosis of TBM were recruited. A definite TBM diagnosis was made when at least one of culture, AFB, PCR (Marais et al., 2010) and Xpert MTB/RIF assay (Patel et al., 2013; Nhu et al., 2014) was positive in the CSF samples. A clinical diagnosis including probable or possible TBM was given based on the diagnostic scoring system (Marais et al., 2010) and response to anti-TBM treatment. Patients with a definite diagnosis of viral, bacterial or cryptococcal meningitis were the mNGS positive controls. A patient with anti-*N*-methyl-D-aspartate (NMDA) receptor (R) encephalitis was a negative control. All TBM and control patients had mNGS tests and traditional tests with their CSF samples from the first lumbar puncture. mNGS analysis usually took 3–4 days. Due to the relatively high cost, mNGS was performed just once for each patient, while traditional tests had been repeated when all previous tests were negative. Brain magnetic resonance imaging (MRI), computed tomography (CT) scan and/or positron emission tomography-computed tomography (PET-CT) were performed for each patient.

### Conventional Tests of CSF

All the traditional tests were performed by Laboratory Medicine Center, Nanfang Hospital or Peking Union Medical College Hospital. Briefly, AFB stain for mycobacteria was detected with AFB kit (BASO, Zhuhai, China) staining the mycolic acid of mycobacterial cell walls (Ellis and Zabrowarny, 1993). The PCR based assay to detect the putative insertion sequence with 123-base pair (bp) length for the MBTC was performed as previously described (Eisenach et al., 1991). Primers were used as: forward primer: 5′-CCTGCGAGCGTAGGCGTCGG-3′, reverse primer: 5′-CTCGTCCAGCGCCGCTTCGG-3′. PCR amplification of *Human herpesvirus 3* (HHV3) was performed as reported (Guan et al., 2016). Alcian blue (BASO, Zhuhai, China) and India ink stains were used to identify the muco substances and polysaccharide capsules of fungi (Zerpa et al., 1996). The cryptococcal antigen enzyme immunoassay (CrAg EIA) was performed to detect the capsular polysaccharide antigens of *Cryptococcus* species complex according to the manufacturer’s instruction (BASO, Zhuhai, China). Anti-NMDAR antibody was analyzed with glutamate receptor kit (EUROIMMUE AG, Luebeck, Germany) according to the manufacturer’s instruction and reference (Cleverly et al., 2014). Briefly, co-transfected HEK293 cells with NR1 and NR2, the subunits of NMDAR, were incubated with patients’ serum or CSF at room temperature for 2 h. Alexa Fluor-labeled secondary antibody was used. Images were obtained by Olympus fluorescence microscopy (Tokyo, Japan).

### mNGS Procedure

DNA extraction, library-preparation and sequencing: Collected CSF samples were snap-froze and stored at −20°C. DNA was extracted from 300 μL CSF sample using the TIANamp Micro DNA Kit (DP316, TIANGEN BIOTECH, Beijing, China) according to manufacturer’s instructions. For samples with suspected fungal infection, we pretreated CSF with glass beads before DNA preparation (Z250465, SIGMA, St. Louis, MO, United States): 500 μL CSF was added into 1.5 ml Eppendorf tube containing equal volume of 0.4–0.5 mm glass beads, vortexed vigorously for 20 min and centrifuged at 8000 rcf for 1 min (Jeon et al., 2014). Then, 300 μL of the supernatant was collected for DNA extraction as described above. DNA libraries were constructed by fragmenting DNA into 200–300 bp, which were flat end-repaired and barcode adapter-ligated; these modified fragments were PCR amplified with BGI reagents (BGI, Tianjin, China). The quality control of the DNA libraries was analyzed with Agilent 2100 equipment (Agilent Technologies, Santa Clara, CA, United States). Qualified libraries with different barcode labeling were pooled together, followed by amplification and enrichment through the Ion OneTouch system (Thermo Fisher Scientific, Waltham, MA, United States). Sequencing were performed at the Ion Torrent NGS platform (BGISEQ-100, BGI, Tianjin, China).

Bioinformatic analysis: Raw sequence data were first filtered by removing low-quality reads to generate clean reads. The clean reads were then mapped to the human database (hg38) to computationally subtract the human sequence using Burrows-Wheeler Alignment software (0.7.10-r789). The remaining data were further classified by simultaneously aligning to four Microbial Genome Databases (Refseq), consisting of viruses, bacteria, fungi, and parasites. The Refseq were downloaded from NCBI^1^, which contains 4152 genome sequences of viral taxa, 3346 bacterial, 206 fungal and 140 parasites genomes or scaffolds related to human infection. After alignment, multiple indicators were comprehensively evaluated to have the list of suspected pathogenic microorganisms output, such as numbers of strictly mapped reads, coverage rate, depth, etc.

### PCR, Sanger and BLAST Validation

The samples with detected sequences mapped to MBTC were further validated with PCR and Sanger (Eisenach et al., 1991). At the same time, the read sequences mapped to MBTC were validated by BLAST searching in the NT database on NCBI online website^2^.

### Interpreting Criteria for Positive mNGS

As reported (Miao et al., 2018), a positive mNGS was given to bacterial and viral cases when the number of reads mapping to a microbe (species level) was 10-fold greater than that of any other microbes. Compared to bacterial and virus, reads aligned to pathogens causing TBM or cryptococcal meningitis were very low due to the low yield of DNA extraction. Thus, when the coverage rate for a fungus (species level) was 5-fold greater than that of another fungi, a positive mNGS was given for the fungus. A positive mNGS for TBM was given when at least one read was mapped to either the species or genus level of MBTC.

### Statistical Analysis

The two-tailed unpaired *t* test was used to analyze whether CSF findings would affect the mNGS results. Paired chi-squire analysis was used to compare the sensitivity and specificity among mNGS and conventional tests. *p*-value < 0.05 was considered significant.

## Results

### Clinical Features of the Participants

**FIGURE 1:**
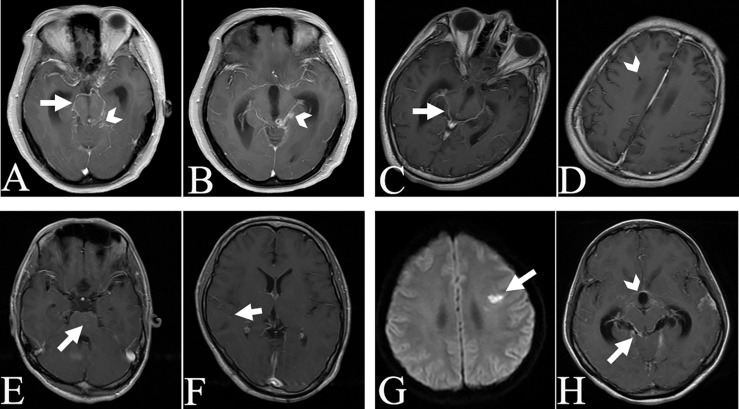
Magnetic resonance imaging images of selected TBM cases. Basicranial meningeal enhancement (arrow) and tuberculoma (arrowhead) in case 4 **(A,B)**. Basicranial meningeal enhancement (arrow) and lateral ventricular drainage tract (arrowhead) in case 5 **(C,D)**. Miliary tuberculous foci (arrow) in case 5 **(E,F)**. Infarction focus (arrow, **G**), meningeal enhancement (arrow, **H**) and enlarged third ventricle of cerebrum (arrowhead, **H**) in case 6.

Among the 23 recruited TBM patients, 12 patients met the criteria for a definite TBM diagnosis based on the classification criteria of TBM (Marais et al., 2010) and evidence of MTB pathogens from at least one of AFB, PCR, culture and Xpert MTB/RIF tests in the CSF samples. Six patients were regarded as probable and five patients as possible TBM as scored in the Supplementary Table S1, and all of these 11 patients responded well to anti-TBM treatment both in clinical and CSF manifestations. As indicated in Table 1, the patients aged from 3 to 73 years with a mean age of 37 years. The symptom duration before admission was 3–150 days. The common neurological manifestations of these patients were headache, epilepsy, delirium, and unconsciousness. Most patients also presented with systemic symptoms such as fever, weight loss, and vomiting. Elevated pressure, increased counts of white cells, low glucose and high protein concentrations in CSF were common findings. Meningeal enhancement (16/23, 69.56%) was the most common cerebral imaging feature of the patients, as shown for example in Figure 1. More than fifty percent of cases (13/23) had evidence of tuberculosis elsewhere by images. None of the patients had received anti-tuberculosis treatment before admission.

**TABLE 1:** Demographic data, CSF findings, imaging feature, and final diagnosis of participants.

**No.**	**Sex**	**Age (y)**	**Sd (d)**	**P**	**Cyto (/uL)**	**R (%)**	**G**	**Pro (g/L)**	**Imaging feature**	**Final diagnosis**
1	F	31	10	285	16	N/C	1.29	1.58	Pulmonary military tuberculosis^∗^; basal meningeal enhancement, tuberculoma^&^	Definite TBM
2	M	44	30	> 330	340	L90	0.41	5.13	Pulmonary military tuberculosis^∗^; hydrocephalus^&^	Definite TBM
3	F	54	13	320	640	L90	1.22	2.09	Active pulmonary tuberculosis^∗^; basal meningeal enhancement, tuberculoma^&^	Probable TBM
4	M	18	30	280	240	L80	1.23	5.47	Hydrocephalus^&^	Possible TBM
5	M	32	50	210	4	N/C	2.8	0.85	Systemic tuberculosis^#^; military tuberculoma^&^	Probable TBM
6	F	29	60	215	0	N/C	1.9	2.02	Pulmonary nodular lesions, suspicious of military tuberculosis^∗^; basal meningeal enhancement, infarct, hydrocephalus^&^	Probable TBM
7	M	48	8	100	12	N/C	5.7	1.88	Basal meningeal enhancement^&^	Definite TBM
8	F	31	10	210	10	N/C	3.07	2.09	Meningeal enhancement, lesions of brain stem^&^	Definite TBM
9	M	22	10	60	0	N/C	2.32	0.34	Meningeal enhancement^&^	Possible TBM
10	M	52	16	260	86	L80	1.34	3.13	Parietal meningeal enhancement^&^	Definite TBM
11	F	33	16	225	86	L70	2.84	> 6	Thoracic spine tuberculosis^∗^; basal meningeal enhancement, tuberculoma^&^	Definite TBM
12	M	58	7	> 330	26	L90	4.45	0.82	Meningeal enhancement^&^	Definite TBM
13	F	45	60	135	34	L80	2.34	0.9	Prior pulmonary tuberculosis^∗^; basal meningeal enhancement, tuberculoma^&^	Definite TBM
14	M	31	7	220	160	L80	0.92	2.06	Prior pulmonary tuberculosis^∗^; meningeal enhancement^&^	Probable TBM
15	F	11	10	200	172	L80	1.39	5.41	Multiple abdominal lymph nodes calcification^∗^	Definite TBM
16	M	35	3	> 330	92.8	N/C	0.74	1.82	Pulmonary military tuberculosis^∗^; meningeal enhancement^&^	Possible TBM
17	F	73	15	260	45	L80	1.5	5.43	Hydrocephalus^&^	Definite TBM
18	F	3	30	140	40	L90	0.74	0.873	Prior pulmonary tuberculosis^∗^; meningeal enhancement^&^	Probable TBM
19	F	42	15	240	80	L90	2.3	3.95	Meningeal enhancement^&^	Possible TBM
20	M	43	3	> 330	353.2	L78	1.23	2.044	Meningeal enhancement^&^	Possible TBM
21	F	40	15	> 330	1	N/C	1.41	1.68	Unremarkable^∗^	Definite TBM
22	M	47	150	225	8	N/C	2.92	0.41	Prior pulmonary tuberculosis^∗^; meningeal enhancement^&^	Probable TBM
23	M	29	120	230	530	L25	1.37	1.24	Systemic tuberculosis^#^	Definite TBM
24	M	41	30	> 330	40	L20	0.64	0.82	Meningeal enhancement^&^	CCM
25	F	64	180	300	250	L25	0.5	1.13	Unremarkable^&^	CCM
26	F	45	1	80	175	L85	3.34	1.63	Parietal meningeal enhancement^&^	BM
27	M	41	30	150	40	L90	2.77	0.89	Hydrocephalus^∗^	BM
28	M	51	1	330	130	L30	7.62	8.72	Unremarkable^∗^	VM
29	M	21	30	130	0	N/C	3.95	0.14	Unremarkable^&^	Anti-NMDAR EC

*No., case number; y, years-old; Sd, symptom duration before admission (days); P, CSF pressure (mmH2O); Cyto, CSF cytology; R, cytological ratio; G, CSF glucose (mmol/L); Pro, CSF protein; F, female; M, male; N/C, not count; L, lymphocyte ratio; N, neutrophile granulocyte; ^∗^, CT scan, ^#^, systemic PET; ^&^, Brain MR plain scan + enhancement; CCM, cryptococcal meningitis; BM, bacterial meningitis; VM, viral meningoencephalitis; NMDAR, *N*-methyl-D-aspartic acid receptor; EC, encephalitis.*

As the mNGS controls, we recruited five patients (No. 24–28) with other infectious meningitis and one patient with auto-immune encephalitis (No. 29). These patients had taken both mNGS and conventional tests. The clinical information of TBM patients and controls were summarized in Table 1.

### Identification of Mycobacterial DNA in CSF Samples by mNGS

Among the 23 TBM cases, stringently mapped reads to MBTC were detected by mNGS in 18 CSF samples (18/23, 78.26%). The number of reads aligned to MBTC were listed in the Table 2. In addition, all stringently mapped reads to MBTC were further validated by BLAST searching in NT database on NCBI online website. The identity of alignment to MBTC was more than 90% in all cases with mNGS positive detection.

**TABLE 2:** Reads of positive mNGS cases.

**Species**	**Reads and case no.**
MBTC	1(19), 2(13) 3(4,5), 7(3,20), 8(2,17), 11(6,15), 12(14), 13(21), 20 (11), 23(16), 30 (23), 43 (1),176 (22), 185(18)
*C. neoformans*	3 (24), 514 (10)
*C. gattii*	5330 (25)
*S. suis*	10140 (26)
*S. pneumoniae*	993 (27)
Human herpesvirus 3	7059 (28)

*MBTC contains five species as follows, *M. tuberculosis*, *M. africanum*, *M. bovis*, *M. canettii*, and *M. orygis.**

The mNGS result of patient 10 was negative with *M. tuberculosis*, but positive with *C. neoformans* (reads, 514, coverage rate, 0.85, and depth, 1). In addition, Alcian blue staining and CrAg EIA test were both positive, confirming the diagnosis of fungal infection. However, the patient did not respond well to anti-fungal treatment and the CSF sample from the 8th lumbar puncture indicated positive AFB stain. Thus, the final diagnosis was a complicated infection of TBM and fungal meningitis.

Control cases 24 and 25 were diagnosed as cryptococcal meningitis based on positive staining of Alcian blue or India ink staining of fungi and CrAg EIA test. The mNGS test found three reads mapping to *C. neoformans* in patient 24 and 5330 reads to *C. gattii* in patient 25. A very high number of unique reads mapped to *Streptococcus suis* in case 26, to *Streptococcus pneumoniae* in case 27, and to HHV3 in case 28. Diagnosis of bacterial meningitis in case 26 or 27 was validated by the positive culture of CSF samples. PCR indicated positive of HHV3 in patient 28, conforming the diagnosis. Patient 29 was diagnosed with anti-NMDAR encephalitis based on confirmation of positive anti-NMDAR antibodies in his serum (1:1 and 1:10) and CSF (1:1), as indicated in the Supplementary Figure S1. No sequence mapping to any pathogen was identified by mNGS in the CSF of the patient. No sequence of MBTC was identified in any control sample, thus, the specificity of mNGS to detect MBTC in this study was 100%.

### Comparison of mNGS Findings With Traditional Methods for the Detection of MTB

We analyzed whether CSF findings would affect the mNGS results by comparing the mNGS positive and negative cases. Cell count numbers (*p* = 0.1530) and protein levels (*p* = 0.2529) did not affect the mNGS results. However, glucose levels significantly correlated the detection rate of mNGS (*p* = 0.0022).

Traditional methods used in the present study included AFB stain, PCR and culture of *M. tuberculosis*. Culture is the gold standard to detect MTB, however, the detect rate is very low. As shown in Table 3, among all patients, only patient 21 was positive from culture of CSF sample, who was also positive for PCR and mNGS tests. Four patients were positive with AFB tests in their first CSF samples (4/22, 18.18%). However, repeated AFB stain increased the total positive detection rate with four more positive cases (8/22, 36.36%). Repeated AFB stain turned out positive at the second repeat (case 11 and 12), 3rd repeat (case 8) and 8th repeat (case 10). Three cases were positive with the PCR method. Unfortunately, Xpert MTB/RIF was available recently and performed in the first CSF samples only in case 17 and 19, with a positive result in case 17. Follow up Xpert MTB/RIF assay in case 15 was positive. Interestingly, mNGS identified sequences mapping to MBTC in the first CSF samples from 18 patients. The agreement of mNGS with four traditional methods including Xpert MTB/RIF assay was 44.44% (8/18). As shown in Table 4, in patients with definite TBM, the negative predictive and positive predictive values of TBM by mNGS were 42.86% and 100%, respectively. The accuracy of mNGS was 77.78%. The sensitivity of mNGS was significantly higher than that of culture (66.67% vs. 8.33%, *p* = 0.023). However, there was no significant difference between mNGS and AFB or PCR (66.67% vs. 33.33%, *p* = 0.13 or 66.67% vs. 25%, *p* = 0.074, respectively). The specificity of each method was 100% and the difference was not significant.

**TABLE 3:** The detection rate among mNGS and conventional methods for all recruited TBM patients.

**Method**	**Positive numbers**	**Numbers without testing**	**Detection rate (%)**
Culture	1^∗^	0	4.35 (1/23)
AFB	8^∧^	1^@^	36.36 (8/22)
PCR	3^[*dollar*]^	4^&^	15.79 (3/19)
mNGS	18^#^	0	78.26 (18/23)

*^∗^, case 21; ^∧^, cases 2, 7, 8 (the 3rd repeat),10 (the 8th repeat), 11 (the 2nd repeats), 12 (the 2nd repeat), 13, 23; ^@^, case 14; ^[*dollar*]^, cases 1, 2, 21; ^&^, cases 14, 18, 15 (Xpert MTB/RIF positive), 17 (Xpert MTB/RIF positive); ^#^, all cases except for 7, 8, 9, 10, and 12.*

**TABLE 4:** Comparison of the sensitivity, specificity, positive predict value, negative predict value, and accuracy among four methods in patients with definite TBM.

**Methods**	**Sensitivity (%)**	**Specificity (%)**	**PPV (%)**	**NPV (%)**	**Accuracy (%)**
Culture	8.33(1/12)	100(6/6)	100(1/1)	35.29(6/17)	38.89(7/18)
PCR	25(3/12)	100(6/6)	100(3/3)	40(6/15)	50(9/18)
1st AFB	33.33(4/12)	100(6/6)	100(4/4)	42.86(6/14)	55.56(10/18)
Acc AFB	66.67(8/12)	100(6/6)	100(8/8)	60(6/10)	77.78(14/18)
1st mNGS	66.67(8/12)	100(6/6)	100(8/8)	60(6/10)	77.78(14/18)

*PPV, positive predict value; NPV, negative predict value; ^∗^Acc, Accumulated test including the first and repeated analysis of CSF samples.*

## Discussion

In the present study, we retrospectively evaluated the feasibility of mNGS for the diagnosis of TBM. We recruited 23 cases with 12 definite TBM and 11 clinical diagnoses of TBM. Comparing different methods in the 1st CSF samples, mNGS reached the highest sensitivity (8/12, 66.67%), followed by AFB stain (4/12, 33.33%), PCR (3/12, 25%) and culture (1/12, 8.33%). Repeated AFB increased positive detection rate to 36.36% (8/22). The agreement of mNGS results with total AFB was 22.22% (4/18), with all conventional methods was 44.44% (8/18). Combination of mNGS and conventional methods increased the detection rate to 95.65%. Our data indicated that mNGS was valuable for the early detection of *M. tuberculosis* DNA in CSF.

Tuberculous meningitis is one of the brain infectious disorders with low detection rate by conventional CSF tests, which is less than 40% (Marais et al., 2010; Nhu et al., 2014). A lot of cases undergo treatment without evidence of pathogens (Lozano et al., 2012; Nhu et al., 2014; Schlaberg et al., 2017; Miao et al., 2018). Although culture is the gold standard for diagnosis, it not only takes time (2–4 weeks) but also has low efficiency (0–40%) (Nhu et al., 2014). There was only one positive culture in our study. In another hand, AFB stain has low sensitivity, but it is universally available, inexpensive, and quick, allowing repeated tests to increase the sensitivity, which was confirmed with four additional positive cases in the present study. Xpert MTB/RIF has formally recommended by WHO in the diagnosis of tuberculosis, the sensitivity of which on TBM diagnosis has been evaluated to be approximately 60% (Patel et al., 2013; Nhu et al., 2014). However, in a recent study with 267 CSF samples, Xpert MTB/RIF assay shows lower sensitivity compared with culture methods (Rufai et al., 2017). Here, among three patients who took the test, two patient were positive. PCR method found three positive in 19 samples, indicating low sensitivity in this study. Among all methods, mNGS had the highest sensitivity (78.26%) comparing the first lumbar puncture data. Our study agreed with other reports that mNGS of CSF is a valuable diagnostic tool for chronic infectious brain diseases, such as neurobrucellosis, neurocysticercosis, fungal meningitis, and TBM (Brown et al., 2018; Fan et al., 2018a, b). Combination of mNGS and traditional methods increased the detection rate to 95.65%. Taken together, our findings suggested that a combination of mNGS with traditional methods could aid in the early diagnosis of TBM.

Notably, we reported a lower number of reads mapping to nucleotides of mycobacteria in most cases (12/18, under 20 for example), compared with the mNGS positive controls or other pathogens reported by other groups (Guan et al., 2016; Yao et al., 2016; Guo et al., 2018). A similar result has been reported by Miao’s study, where at least one read mapping to MBTC is regarded as mNGS positive (Miao et al., 2018). Detection of mycobacteria may be complicated because mycobacteria are intracellular pathogens with very low abundance in CSF. Due to the low abundance of MTB and low possibility of contamination (Doughty et al., 2014; Miao et al., 2018; Simner et al., 2018), the cut-off value for MTB detection should be set low. In the present study, at least one read mapping to MBTC was regarded as positive (Miao et al., 2018).

Case 10 was a complicated infection of TBM and cryptococcal meningitis. The patient was admitted with headache, sudden onset of deafness and fever for 16 days. The mNGS result and traditional tests from the first lumbar puncture agreed with the diagnosis of meningitis with *C. gattii*. After anti-fungal treatment for 8 days, cytological analysis of CSF revealed a remarkable decrease in lymphocytes, but CSF pressure increased, with an accompanying decrease in glucose and an increase in protein levels. The patient suffered from recurring headache and worsening neck stiffness. After another 22 days, AFB stain was positive at the 8th lumbar puncture. Anti-tuberculosis treatment was immediately added in addition to anti-fungal treatment. The patient gradually recovered except for his deafness.

As other studies have reported (Guan et al., 2016; Yao et al., 2016), contaminated or background microbe genes were detected in each sample. The most frequently detected bacteria were *Propionibacterium acnes*, followed by *Acidovorax KKS102* and *Brevundimonas subvibri-oides*. Because of the high sensitivity of mNGS technique, microbes from the environment source, skin flora or the reagents could contribute to contamination (Brown et al., 2018). Therefore, clinicians should be cautious when interpreting the results of mNGS alone.

There were several limitations in this study. First, mNGS was done only once for each patient due to the cost of the procedure (3100 RMB, less than 500 dollars), which restricted its widespread use and duplicated detection. Since the time of CSF collection for mNGS may differ from the peak time of pathogens occurrence in CSF, repeated mNGS tests might improve the sensitivity. In addition, due to the low reads of MBTC in most cases, mNGS alone does not necessarily have advantages over traditional methods. Second, the sample size was relatively small. In our study, there was no significant difference between mNGS and AFB or PCR. Further investigation with larger samples would be warranted to evaluate the diagnostic value of mNGS in TBM.

## Conclusion

In conclusion, comparison of mNGS with conventional tests in CSF from 23 patients with TBM indicated that mNGS (78.26%) was the most sensitive method to detect mycobacteria DNA in this study. mNGS might be used as a front-line diagnostic tool for TBM diagnosis and a combination of mNGS and conventional methods would maximize the detection rate.

## Author Contributions

YFH, SP, and HG designed the study. SW, KH, and YFH wrote the manuscript. SW, DW, YW, DZ, JZZ, HX, HJ, JZ, HG, and SP attended patients and provided clinical data. YC, XL, and YWH organized and prepared the CSF samples. WL and GL performed the AFB and Alcian blue staining. SW provided magnetic resonance imaging data. YG, RS, YL, and XN performed next generation sequencing and data analysis. All authors read and approved the final version of the manuscript.

## Conflict of Interest Statement

YG, RS, and XN were employed by Tianjin Medical Laboratory, BGI-Tianjin, BGI-Shenzhen, Tianjin, China. YL was employed by the BGI Genomics, BGI-Shenzhen, Shenzhen, China. The remaining authors declare that the research was conducted in the absence of any commercial or financial relationships that could be construed as a potential conflict of interest.

## Footnotes

1 https://www.ncbi.nlm.nih.gov/genome/

2 https://blast.ncbi.nlm.nih.gov/Blast.cgi?PROGRAM=blastn&PAGE_TYPE=BlastSearch&LINK_LOC=blasthome

## Supplementary Material

FIGURE S1 Detection of anti-NMDAR antibody in patient 29. Green fluorescence indicated anti-NMDAR antibody positive. CSF with a titer of 1:1 showed positive, sera with titer of 1:1 and 1:10 showed positive. CSF negative and positive controls for anti-NMDAR encephalitis were provided by the kit.

TABLE S1 The diagnostic score of probable and possible TBM patients.
