# Supplementary figures and images for: The Feasibility of Metagenomic Next-Generation Sequencing to Identify Pathogens Causing Tuberculous Meningitis in Cerebrospinal Fluid
Source: Front Microbiol. 2019 Sep 3;10:1993. doi: 10.3389/fmicb.2019.01993 (PMC6733977; doi:10.3389/fmicb.2019.01993)

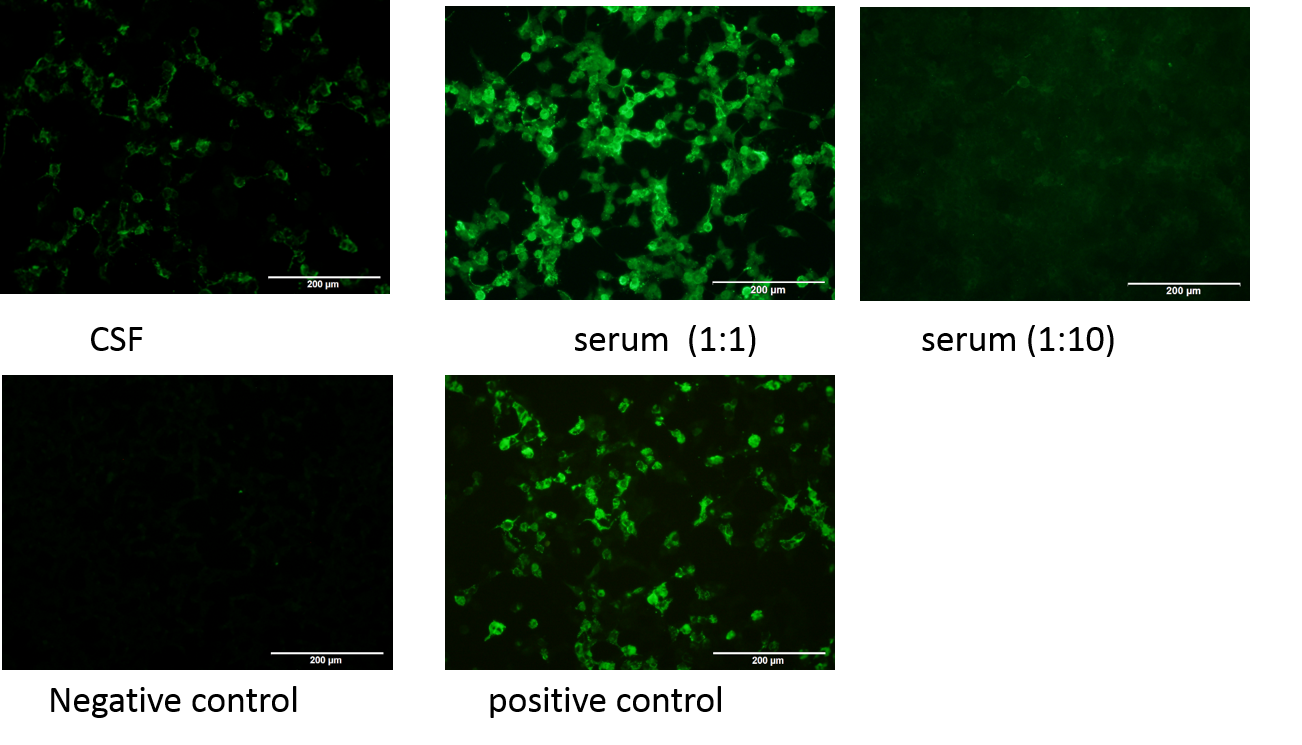

Supplement: FIGURE S1 — Detection of anti-NMDAR antibody in patient 29. Green fluorescence indicated anti-NMDAR antibody positive. CSF with a titer of 1:1 showed positive, sera with titer of 1:1 and 1:10 showed positive. CSF negative and positive controls for anti-NMDAR encephalitis were provided by the kit. [file Image_1.TIF]
