# Supplementary material for: The Feasibility of Metagenomic Next-Generation Sequencing to Identify Pathogens Causing Tuberculous Meningitis in Cerebrospinal Fluid
Source: Front Microbiol. 2019 Sep 3;10:1993. doi: 10.3389/fmicb.2019.01993 (PMC6733977; doi:10.3389/fmicb.2019.01993)
Supplement: TABLE S1 — The diagnostic score of probable and possible TBM patients. [file Table_1.DOCX]

**Supplementary table 1** The diagnostic score of probable and possible TBM patients

| No. | Clinical criteria | CSF criteria | Cerebral imaging criteria | Evidence of tuberculosis elsewhere | Score for TBM |
| --- | --- | --- | --- | --- | --- |
| 3 | 5 | 4 | 4 | 4 | 17 |
| 4 | 5 | 4 | 1 | 0 | 10 |
| 5 | 6 | 1 | 2 | 4 | 13 |
| 6 | 6 | 3 | 4 | 4 | 17 |
| 9 | 5 | 1 | 2 | 0 | 8 |
| 14 | 4 | 4 | 2 | 4 | 14 |
| 16 | 0 | 4 | 2 | 4 | 10 |
| 18 | 4 | 4 | 2 | 4 | 14 |
| 19 | 4 | 4 | 2 | 0 | 10 |
| 20 | 0 | 4 | 2 | 0 | 6 |
| 22 | 6 | 1 | 2 | 4 | 13 |
